# Determining the position of the lingula and the mandibular foramen using the antilingula in orthognathic surgery

**DOI:** 10.1186/s12903-024-04286-7

**Published:** 2024-04-27

**Authors:** Chakorn Vorakulpipat, Tawepong Arayapisit, Pee Topothai, Vathanai Bhunyanaphakul, Keerati Tiptimaphan, Nattha Apilakkitakul, Varunya Chantadul

**Affiliations:** 1 Department of Oral and Maxillofacial Surgery, Faculty of Dentistry, Mahidol University, Bangkok, Thailand; 2 Department of Anatomy, Faculty of Dentistry, Mahidol University, Bangkok, Thailand; 3 Department of Oral Medicine and Periodontology, Faculty of Dentistry, Mahidol University, Bangkok, Thailand; 4 Department of Prosthodontics, Faculty of Dentistry, Mahidol University, Bangkok, Thailand; 5 Sirindhorn College of Public Health, Suphanburi, Thailand; 6 Dentineer Dental Clinic, Bangkok, Thailand

**Keywords:** Antilingula, Lingula, Mandibular foramen, Orthognathic surgery

## Abstract

**Background:**

The antilingula located on the lateral surface of the mandibular ramus has been served as a surgical landmark for the mandibular foramen on the medial surface for decades. However, whether the antilingula truly represents the lingula which is the bony prominence overlapping the mandibular foramen, or the foramen itself, is still unclear. This study thus aimed to examine the position of the antilingula in relation to three reference points: the lingula, the anterior and the posterior borders of the mandibular foramen, as well as to the reference plane used in the inferior alveolar nerve block, and to the posterior border of the mandible.

**Methods:**

This observational study was performed in 113 Thai dry mandibles. The antilingula were identified followed by transferring the reference points to the lateral surface. The distances from the antilingula to the reference points, the reference plane and the posterior border of the ramus were then measured. Chi-square test was calculated for side-dependency of the antilingula. Paired t-test was calculated for difference in measurements in left and right sides.

**Results:**

The antilingula could be identified in 92.48% of the mandibles with 86.67 – 90.00% accuracy and 86.67% reliability. There was no significant difference in the presence of the antilingula on left and right sides (*p* = 0.801). Only 2.5% and 0.83% of the antilingula correspond to the lingula and the anterior border of the mandibular foramen, respectively. However, 85% of the reference points were located within 11 mm radius. The antilingula was found located 2.80 mm inferior to the reference plane and 16.84 mm from the posterior border of the ramus.

**Conclusions:**

The antilingula does not concur with the reference points on the medial surface. Our study also suggests that the safe area for vertical osteotomy is 11 mm posterior to the antilingula or at 30% of the length from the posterior border parallel to the occlusal plane. The use of more accurate techniques in localizing the mandibular foramen combined with the antilingula is more recommended than using the antilingula as a sole surgical guide.

## Background

Correction of mandibular deformities may involve orthodontic treatment and orthognathic surgery, or either one, depending on the patient’s consent and the doctor’s expertise. The modern mandibular osteotomy was first described in 1942 by Schuchardt [1] and has been continuously modified by several groups of surgeons to establish the safer procedures with less complications [2–7]. Currently, the common surgical techniques for splitting and repositioning a segment of the mandible are bilateral sagittal split ramus osteotomy (BSSRO) and intraoral vertical ramus osteotomy (IVRO) [8–10]. Although both procedures are often successful [11, 12], an osteotomy along the medial surface of the ramus might increase risks of excessive bleeding or permanent paresthesia of the lower jaw due to injury to the inferior alveolar neurovascular bundle (IANB) entering the mandibular foramen [11–13]. The bony anatomical landmark for determining the safe zone of osteotomy on the lateral surface of the ramus is thus required to prevent these unfavorable outcomes.

The antilingula (AL) is a small prominence on the lateral surface of the ramus of the mandible. This structure was initially marked as a surgical reference for the mandibular foramen on the medial surface by Caldwell and Letterman [2]. Since then, several postulates regarding the position of the AL in relation to other anatomical structures have been established. Some surgeons hypothesized that the location of the AL roughly corresponded to the true lingula (LG) which is a bony projection overlapping the mandibular foramen [2, 14–16] and the compression from the nerves and blood vessels entering the foramen may cause the elevation on the opposite surface [17]. Others suggested that the AL possibly related to the insertion of the masseter muscle [18, 19]. In spite of being commonly used as a bony landmark for orthognathic surgery for several decades [9], whether the AL correctly represents the position of the LG or the opening of the mandibular canal is still under debate. Therefore, this study aimed to determine the position of the AL in relation to the LG, the anterior and the posterior borders of the mandibular foramen in Thai dry mandibles. In addition, the position of the AL was also determined in relation to the reference plane used in the inferior alveolar nerve block, and the posterior border of the ramus to provide a surgical guide during vertical osteotomy.

## Materials and methods

### Ethics

Exemption from ethics approval was granted by the Faculty of Dentistry and the Faculty of Pharmacy, Mahidol University, Institutional Review board (MU-DT/PY-IRB), reference number: COE.No.MU-DT/PY-IRB 2021/022.2206. All experiments were performed according to the ethical principles of the Declaration of Helsinki.

### The presence of the AL

One hundred thirteen Thai dried mandibles of unknown age and sex from the Department of Anatomy, Faculty of Medicine, Siriraj Hospital and the Department of Anatomy, Faculty of Dentistry, Mahidol University were initially selected. The most protruding point of the AL prominence on the ramus was identified as the AL point, while the most superior point of LG was identified as the LG point. All reference points were marked by the expert (the oral and maxillofacial surgeon) using an ultraviolet (UV) marker.

### Accuracy and reliability in identifying the AL

The AL from 15 mandibles were identified by 3 dental students using an UV marker and re-examined by the expert using UV light. The locations of the AL identified by the students were compared to the expert and calculated as percent accuracy. The locations were also compared among 3 students and calculated as percent reliability.

### Selection criteria for the mandibles

For the following experiments, the mandibles with these criteria were included in this study: presence of the AL in both sides of the ramus and presence of at least a premolar and the first molar (excluding the third molar).

All measurements were executed in triplicate using an 8-inch ABSOLUTE digimatic caliper (Mitutoyo, Japan) before being averaged.

### The position of the reference points in relation to the AL in the x–y axis

**Fig. 1:**
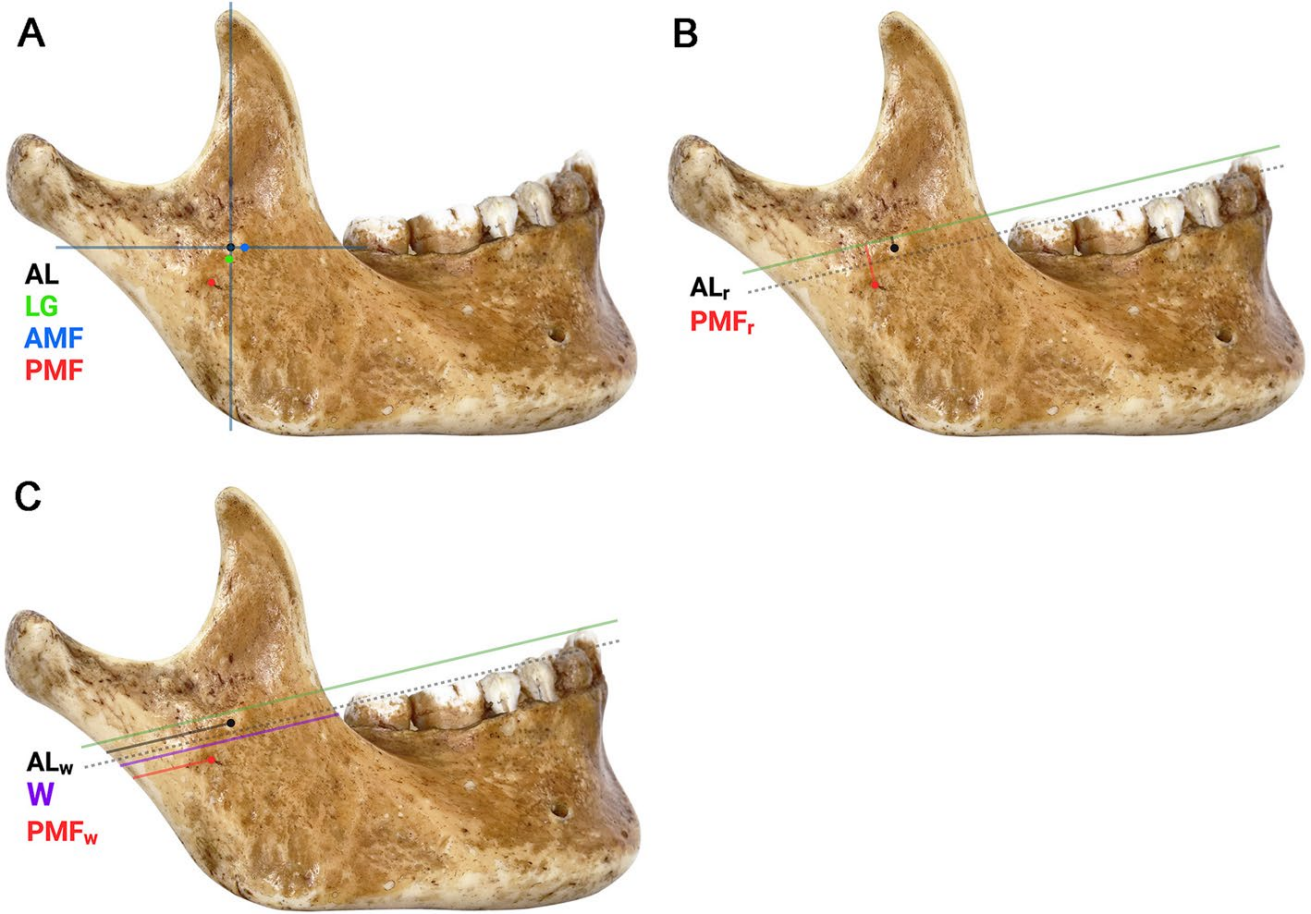
The position of the AL and the reference points. **A** The positions in relation to the x and y axes (blue lines). **B** The positions in relation to the occlusal plane. **C** The positions in relation to the posterior border of the ramus of the mandible. The green lines represent the reference plane which was parallel to the occlusal plane and passed the most concave point of the anterior border of the ramus (coronoid notch). The dashed lines represent the occlusal plane

The LG, the anterior border of the mandibular foramen (AMF) and the posterior border of the mandibular foramen (PMF) were set as the reference points and transferred to the lateral surface of the ramus using a 6-inch Outside Spring Caliper (Solar, India). The caliper was placed perpendicular to the surface of ramus and its axis was parallel to the horizontal plane. The distances from the reference points were measured by a digimatic caliper in relation to the AL which was set as an origin (0,0) and recorded as a co-ordinate where x was parallel to a horizontal axis, y was parallel to a vertical axis (Fig. 1A).

### The position of the AL and the PMF in relation to the reference plane

The PMF was transferred to the lateral surface as previously described and the most concave point of the anterior border of the ramus (the coronoid notch) was marked. The occlusal plane in our study was marked by the line that passed the buccal cusp of a premolar and the mesiobuccal cusp of the first molar. The reference plane was determined as the plane which was parallel to the occlusal plane and passed through the coronoid notch. The distance from the AL and the PMF to the reference plane (AL_r_ and PMF_r_, respectively) were measured perpendicular to the plane (Fig. 1B). The narrowest width of the ramus in an antero-posterior direction (W), the distance from the posterior border of the ramus to the AL and the PMF (AL_w_ and PMF_w_, respectively) were measured parallel to the reference plane (Fig. 1C). The ratios of AL_w_/W and PMF_w_/W were subsequently calculated to normalize variations in size among the mandibles. 

### Statistical analysis

The side-dependency of the presence of the AL was analyzed using chi-square test. The statistical difference in measurements in left and right sides were analyzed using paired t-test. The measurements were compared to zero using one sample t-test. All the tests were performed using IBM SPSS Statistics for Windows, version 27.0 (IBM Corp; Armonk, NY, USA). The distributions of all reference points from the AL were analysed using Minitab 17.0 (Minitab, Inc; State College, PA, USA) and the scatter plots were generated using GraphPad Prism 10.0 (GraphPad Software, Inc. San Diego, CA, USA).

## Results

### The presence of the AL

Of 113 Thai mandibles (226 sides), the AL was identified in 209 sides (92.48%) where 92.04% was on the left side and 92.92% was on the right side. The presence of the AL in both sides was observed in 102 mandibles (90.27%). There was no significant difference in the presence of the AL on left and right sides (*p* = 0.801).

### Accuracy and reliability in identifying the AL

The locations of the AL in 30 sides of the mandibles identified by 3 students were confirmed by the expert. The accuracy of identification was in the range of 86.67 – 90.00% (Table 1). Comparison of the AL location was also performed among the students to assess the reliability of identification which showed that 86.67% of the locations were agreed by all students whereas 13.33% were agreed by 2 students (Table 1).

**Table 1:** The percentages of accuracy and reliability in identifying the AL

		**Accuracy**			**Reliability**	
		Student 1	Student 2	Student 3	Partial agreement	Total agreement
**%**		90.00	90.00	86.67	13.33	86.67
**95% CI**	**Upper**	97.89	97.89	96.24	30.72	96.24
	**Lower**	73.47	73.47	69.28	3.76	69.28

After identifying the AL, 60 mandibles were selected from 113 mandibles based on the inclusion criteria to study the relationships between the AL and other structures.

### The position of the AL in the x–y axis

 The reference points (LG, AMF and PMF) were transferred to the lateral surface of the ramus and their distance from the AL were measured. There was no significant difference in the positions between left and right sides (Table 2). Therefore, the data from both sides were averaged and included in the analysis.

**Table 2:** Relationships between the reference points (LG, AMF and PMF) and the antilingula

Variables	Mean ± SD		*p*-value	Mean ± SD	*p*-value
	**Left**	**Right**		**Total**	
LG in the horizontal plane (LG_x_)	0.01 ± 2.84	0.30 ± 2.53	0.420	0.15 ± 2.68	0.528
LG in the vertical plane (LG_y_)	-1.75 ± 3.54	-1.40 ± 2.97	0.419	-1.58 ± 3.26	0.000*
AMF in the horizontal plane (AMF_x_)	1.01 ± 3.20	1.78 ± 2.75	0.101	1.40 ± 2.99	0.000*
AMF in the vertical plane (AMF_y_)	-0.14 ± 2.78	-0.06 ± 2.82	0.826	-0.10 ± 2.79	0.689
PMF in the horizontal plane (PMF_x_)	-1.73 ± 3.51	-2.56 ± 2.99	0.055	-2.15 ± 3.27	0.000*
PMF in the vertical plane (PMF_y_)	-7.98 ± 3.02	-7.25 ± 3.30	0.083	-7.61 ± 3.17	0.000*

^*^ Statistical significance at *p* < 0.05 by one sample t – test

### The position of the LG in relation to the AL

In the x-axis, the most posterior and anterior LGs were 6.16 mm and 9.38 mm, respectively, from the AL. However, there was no statistically significant difference between the position of the LG and the AL in antero-posterior dimension, with the LG being located 0.15 ± 2.68 mm anterior to the AL (Table 2). Conversely, in the y-axis, the position of the LG was 1.58 ± 3.26 mm inferior to the AL with the range of 11.00 mm inferior to the AL and 7.92 mm superior to the AL (Table 2). At 95% confidence interval, the LG was 0.99 – 2.16 mm inferior to the AL.

**Fig. 2:**
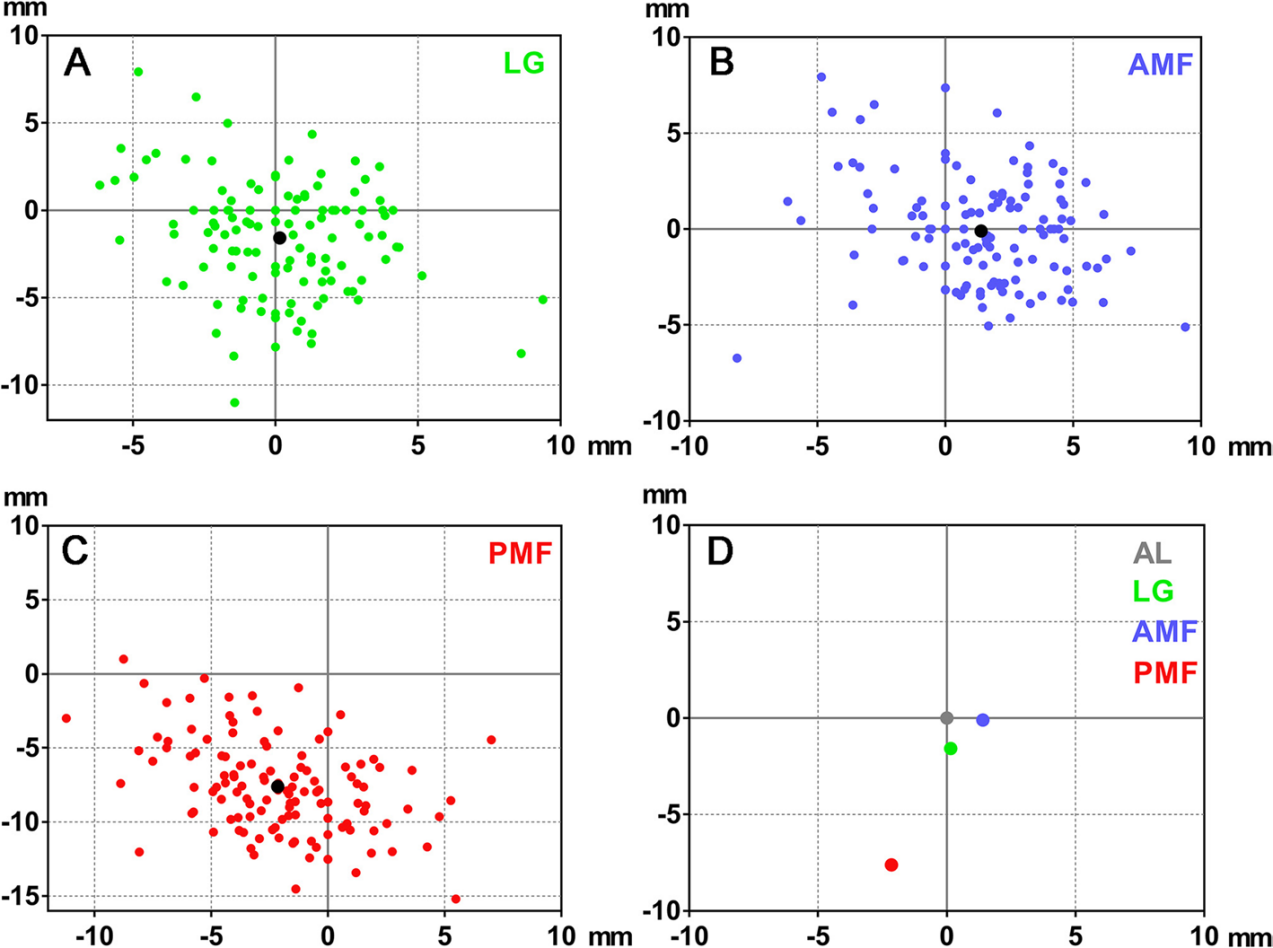
Scatterplot showing the distributions of the reference points in relation the AL (0,0). **A** The LG. **B** The AMF. **C** The PMF. Black dot; average position of each reference point. **D** All average reference points in relation to the AL

The analysis of the distribution of the LG from the AL showed that 31.67% of the LG was located antero-inferior to the AL (Fig. 2A) and 68.33% scattered within 5 mm radius from the AL while 86.67% scattered within 6 mm (Table 3).

**Table 3:** Distribution of the lingula (LG), the anterior border of the mandibular foramen (AMF) and the posterior border of the mandibular foramen (PMF) within a 10 mm radius from the AL

Radius (mm)	*N* (%)		
	**LG**	**AMF**	**PMF**
**0**	3 (2.50%)	1 (0.83%)	0 (0.00%)
**0 < x ≤ 5**	79 (65.83%)	88 (73.33%)	8 (6.67%)
**5 < x ≤ 6**	22 (18.33%)	15 (12.50%)	7 (5.83%)
**6 < x ≤ 7**	6 (5.00%)	8 (6.67%)	10 (8.33%)
**7 < x ≤ 8**	5 (4.17%)	5 (4.17%)	20 (16.67%)
**8 < x ≤ 9**	1 (0.83%)	0 (0.00%)	21 (17.50%)
**9 < x ≤ 10**	1 (0.83%)	1 (0.83%)	16 (13.33%)
** > 10**	3 (2.50%)	2 (1.67%)	38 (31.67%)
**Total**	120 (100.00%)	120 (100.00%)	120 (100.00%)

### The position of the AMF in relation to the AL

In the x-axis, the position of the AMF was 1.40 ± 2.99 mm anterior to the AL with the range of 8.14 mm posterior to the AL and 9.38 mm anterior to the AL (Table 2). At 95% confidence interval, the AMF was 0.86 – 1.94 mm anterior to the AL. In the y-axis, the most inferior and superior positions of AMF were 6.73 mm, and 7.92 mm, respectively, from the AL. However, there was no statistically significant difference between the position of the AMF and the AL in supero-inferior dimension, with the AMF being located 0.10 ± 2.79 mm inferior to the AL (Table 2).

The analysis of the distribution of the AMF from the AL showed that 39.17% of the AMF was located antero-inferior to the AL (Fig. 2B) and the majority of the AMF positions (86.66%) scattered within 6 mm radius from the AL (Table 3).

### The position of the PMF in relation to the AL

The PMF was in different position from the AL in both x- and y- axes. In the x-axis, the position of the PMF was 2.15 ± 3.27 mm posterior to the AL with the range of 11.21 mm posterior to the AL and 7.00 mm anterior to the AL (Table 2). At 95% confidence interval, the PMF was 1.55 – 2.74 mm posterior to the AL. In the y-axis, the position of the PMF was 7.61 ± 3.17 mm inferior to the AL with the range of 15.21 mm inferior to the AL and 1.00 mm superior to the AL. At 95% confidence interval, the PMF was 7.04 – 8.19 mm inferior to the AL (Table 2).

The analysis of the distribution of the PMF from the AL showed that the majority of the PMF (74.17%) was located postero-inferior to the AL (Fig. 2C) and only 6.67% scattered within 5 mm radius from the AL, while 55.00% scattered within 9 mm and 80.83% scattered within 11 mm radius (Table 3).

The relationship between the AL and all reference points is demonstrated in Fig. 2D.

### The position of the AL in relation to the reference plane

The distances from the AL and the PMF to the reference plane were measured perpendicular to the plane (Table 4). The AL was located 2.80 ± 3.69 mm inferior to the reference plane (AL_r_). The furthest distances of the AL from the plane were 11.14 mm inferiorly and 17.00 mm superiorly. The PMF was located inferior to the plane with the distance of 8.36 ± 4.45 mm (PMF_r_). The furthest distances of the PMF from the plane were 17.55 mm inferiorly and 17.17 mm superiorly.

**Table 4:** Relationships between the anatomical landmarks (AL and PMF) and the reference plane

Variables	Mean ± SD		*p*-value	Mean ± SD	*p*-value
	**Left**	**Right**		**Total**	
Distance from the AL perpendicular to the reference plane (AL_r_)	-1.88 ± 3.96	-3.71 ± 3.17	0.004^#^	-2.80 ± 3.69	0.000*
Distance from the PMF perpendicular to the reference plane (PMF_r_)	-8.31 ± 4.49	-8.41 ± 4.46	0.900	-8.36 ± 4.45	0.000*
Distance from the AL to the posterior border of the mandibular ramus (AL_w_)	16.42 ± 2.73	17.26 ± 2.40	0.017^#^	16.84 ± 2.60	-
Distance from the PMF to the posterior border of the mandibular ramus (PMF_w_)	10.41 ± 2.43	10.95 ± 3.97	0.091	10.68 ± 2.72	-
The narrowest width of the ramus (W)	33.85 ± 3.63	34.22 ± 3.93	0.314	34.03 ± 3.77	-

^#^Statistical significance at *p* < 0.05 by paired t – test

^*^Statistical significance at *p* < 0.05 by one sample t – test

The AL and the PMF were also measured from the posterior border of the mandible parallel to the reference plane (AL_w_ and PMF_w_, respectively, Table 4). The AL_w_ distance was 16.84 ± 2.60 mm with the range of 10.94 – 23.34 mm and the PMF_w_ distance was 10.68 ± 2.72 mm with the range of 4.55 – 18.98 mm. The narrowest width measured parallel to the plane (W) was 34.03 ± 3.77 mm with the range of 24.43 – 46.46 mm thereby giving the AL_w_/W and PMF_w_/W ratios of 0.50 and 0.31, respectively.

## Discussion

The AL is a bony prominence on the lateral surface of the mandibular ramus and has been used as a landmark during orthognathic surgery for decades [2, 20, 21]. However, the presence of the AL has been shown to vary across studies, even within those focused on Asian populations, ranging from 32.02 – 100% as demonstrated in Table 5. The methods of study probably impacted the identification of the AL, as it tends to be less visible in CT images compared to dry mandibles. In this present study, the AL could be detected in most samples and the presence of the AL was not shown to be associated with the presence of the LG. Our findings were consistent with the study of Apinhasmit [22] which was performed in Thai mandibles, albeit higher occurrence, and in agreement with Park et al. (Korean mandibles) [23] and Hsiao (Taiwanese patients) [24].

**Table 5:** Presence and reliability of the AL

	**Year of study**	**Country of study**	**Method of study**	**N (sides)**	**Occurrence** **(%)**	**Reliability** **(%)**
Yates et al. [16]	1976	USA	Dry mandibles	140	44	59
Langston & Tebo [31]	1977	USA	Dry mandibles	100	100	-
Martone et al. [38]	1993	USA	Dry mandibles	63	42	-
Pogrel et al. [15]	1995	USA	Dry mandibles	40	100	22.5
Aziz et al. [26]	2007	USA	Dry mandibles	36	100	-
Apinhasmit et al. [22]	2011	Thailand	Dry mandibles	184	80.4	-
Monazzi et al. [39]	2012	Brazil	Dry mandibles	88	65.9	-
Park et al. [40]	2014	Korea	CT images	250	45.5	-
Hosapatna et al. [14]	2015	India	Dry mandibles	100	53	-
Park et al. [23]	2018	Korea	Dry mandibles	40	100	-
Zhao et al. [41]	2019	China	CT images	204	45	-
Hsiao et al. [24]	2020	Taiwan	CT images	180	81.10	-
Kapur et al. [25]	2021	UK^a^	Dry mandibles	480	100	59.15^b^
Sinanoglu et al. [42]	2023	Turkey	CT images	228	32.02	-
Present study	2024	Thailand	Dry mandibles	226	92.48	86.67

^a^The mandibles from different ethnic groups were included in this study

^b^The average of the horizontal and the vertical planes

In orthognathic surgery, especially IVRO and inverted-L osteotomy (ILO) techniques, locating the LG from the buccal side of the mandibular ramus is a challenging task. Therefore, it is of importance that the AL, the anatomical landmark indicating the LG position on the lateral side, should be clearly visible and reliable. The accuracy in identification of the AL was assessed by comparing the positions identified by each dental student and the expert. The reliability was also evaluated by comparing the position identified by 3 students. Our assessments showed 86.67 – 90.00% accuracy and 86.67% reliability in identification of the AL which are higher than the studies of Pogrel [15], Yates [16] and Kapur [25] (Table 5). The high accuracy and reliability in our study supports the practicality of this landmark since it could be detected by both novices and experts without difficulty. Nevertheless, owing to the differences in reliability and accuracy among different ethnic groups, multi-center or multi-ethnicity studies should be warranted to confirm our findings before generalizing to other populations.

Although the AL on the lateral surface of the mandible has been commonly used to approximate the LG on the medial surface, several lines of evidence have proven that the AL was not exactly located on the same position as the LG [15, 22, 25, 26]. Indeed, the recent study indicated no correlation between these structures [24]. Our study showed that only 2.5% of the LG exactly corresponded to the AL and 68.33% distributed within 5 mm radius while 86.67% were found within 6 mm radius from the AL. However, while the common position of the LG was shown to be posterior and inferior to the AL [15, 22, 26, 27], the majority of the LG in this present study were located inferior to the AL with greater propensity in the anterior region, giving the percentage of 31.67%. The distribution in this antero-inferior region is comparable to the study of Apinhasmit (27.00%) [22]. Considering the distribution from the AL (Table 6), Pogrel [15] and Apinhasmit [22] showed 43.3% and 84.50%, respectively, of the LG were located within 5 mm radius. The latter is consistent with the distribution within 6 mm in our study. Because the difference of 1 mm is unlikely to be significant in clinical practice, it can be assumed that the LG are mostly located inferior to the AL and within 5 – 6 mm radius in Thai mandibles.

**Table 6:** Distribution of the LG within 10 mm radius (5 mm intervals) from the AL compared between studies

	**N (sides)**	**0 mm (%)**	**Within 5 mm (%)**	**Within 5**–**10 mm (%)**	**More than 10 mm (%)**
Pogrel et at. [15]	40	17.5	43.3	50.0	6.7
Apinhasmit et al. [22]	148	0.0	84.5	15.5	0.0
Present study	120	2.50	68.33	29.17	2.50

Our study demonstrated that the LG was not always located on the same position as the AL on the lateral side. Moreover, the morphology of the LG has been shown to be diverse among genders and racial groups [28, 29]. Tuli et al. [30] classified such variations into 4 types: truncated, triangular, nodular and assimilated. Due to the diversity of its morphology, using the AL may not be the most suitable approach to identify the LG. Therefore, we also considered the relationship between the posterior border of the mandibular foramen which is another anatomical structure relating to the IANB and the AL. Our result is consistent with the previous studies [16, 23, 31] that the mandibular foramen was usually located posterior and inferior to the AL. Regarding its distribution, Yates et al. found 37.10% of the foramen was within 5 mm radius [16] from the AL whereas only 6.67% were detected in our study. This discrepancy might be because the foramen in the study of Yates et al. referred to the deepest point of the foramen. It is important that the size of the foramen should be taken into account as the width, which was shown to vary from 3 to 11 mm, is used to determine of the length of the horizontal osteotomy in ILO and BSSRO [32]. Consequently, the anterior border of the foramen was also investigated. Our result indicated that the AMF did not represent the position of the AL which is in agreement with the previous report by Hosapatna et al. [14].

We demonstrated that the mandibular foramen did not correspond to the AL especially its posterior border which was located further than other reference points from the AL. Therefore, the speculation that the IANB compresses the medial surface of the ramus and leads to a prominence on the lateral surface when the bundle passes through the foramen may not hold true. Reitzel et al. [18] and Hogan and Ellis [17] proposed another hypothesis that the AL is a bony elevation occurring in response to the tendon of the deep head of masseter muscle which inserts at the midpoint of the ramus. The elevation was called the masseteric apical bump [18, 19]. In addition, the bony ridge similar to that usually found at the insertion of a muscle was clearly observed in the mandible of primates and canines at the same area as the masseteric apical bump in human mandible [17]. This assumption is thus more likely to underlie the cause of the AL.

Apart from the horizontal plane, we also investigated the location of the reference points in relation to the reference plane which was parallel to the occlusal plane and passed through the coronoid notch, or the plane that serves as a guide for inferior alveolar nerve block. This reference plane is assumed to be at the level of the mandibular foramen and lies approximately 6–10 mm superior to the occlusal plane [33]. Our finding showed that the AL and the PMF were inferior to the reference plane with the distances of 2.80 ± 3.69 mm and 8.36 ± 4.45 mm, respectively. Since our results also indicated that the LG was 1.58 ± 3.26 mm inferior to the AL, we could estimate that the LG was positioned at 4.5 mm inferior to the reference plane, or approximately 5.5 mm superior to the occlusal plane. Consistent with our data, a previous study on Thai mandibles reported that 78.52% of the LG was located 4.5 mm superior to the occlusal plane [29]. To our knowledge, our study is the first to report the AL and the PMF relative to the plane used in the inferior alveolar nerve block, a plane well recognized by most dental surgeons. These relationships can be applied to estimate the position of the irregular shaped AL and the posterior border of the mandibular foramen when the AL cannot be identified.

The recommended distance to avoid damage to the IANB in IVRO is 7 – 8 mm anterior to the posterior border of the ramus [9] or 8 mm posterior to the AL [25]. In addition, Werther and Hall [34] proposed that the length of the ramus posterior to the osteotomy line should be at least 6 mm to preserve the viability of the remaining bone. When the AL is used as a reference point, our results suggested that in Thai mandibles, the distance of 10 – 11 mm posterior to the AL is considered safe for the vertical osteotomy. This distance is far enough to avoid IANB while the bone viability can still be preserved. Due to the variations in the size of the ramus, the ratios of the measurements were also calculated. Our PMF_w_/W ratio is also consistent with Park et al. and Chen et al. [23, 35] indicating that the region suitable for IVRO is the point at the posterior one third of the ramus, or at 30% of the horizontal length of the ramus from the posterior border. However, the location of the AL and the mandibular foramen can vary among different facial morphologies i.e. short and broad face, or long and narrow face, and skeletal patterns i.e. skeletal class I, II and III [10, 36]. Therefore, careful considerations should be taken when using the AL as a sole reference point of the mandibular foramen. The estimation of the mandibular foramen in relation to the AL using a cone beam computed tomography (CBCT) can also endorse the use of the AL in clinical setting [27, 35].

The limitation of this study was that the mandibles were of unknown age and sex which are the factors affecting the morphology of the mandibular ramus and the structures on the ramus. A recent study showed that the location of the AL and the dimensions of the ramus were correlated with sex [10], probably due to the smaller size of female mandibles compared to male mandibles. Another study also indicated the tendency for the mandibular foramen to shift superiorly with advancing age [37]. Nevertheless, whether the location of the AL changes in a similar pattern with the foramen requires further clarifications. In clinical practice, the ages of patients undergoing orthognathic surgery are generally younger than those of the dried mandible in our study. This age difference should be considered when estimating the location of the mandibular foramen.

## Conclusions

The AL is an anatomical structure which can be identified in most of the Thai mandibles with high accuracy and reliability. Although the AL is not located on the exact position of the LG, the anterior or the posterior borders of the mandibular foramen on the medial surface, its position can be used as a reference point to estimate these structures which are predominantly located within 11 mm radius. Based on the occlusal plane and the posterior border of the ramus, this present study suggested the safe region for vertical osteotomy which is 10–11 mm posterior to the AL or at 30% of the horizontal length from the posterior border. The use of AL to approximate the mandibular foramen during operation should be combined with the distance determined from the CBCT during preoperative planning to prevent iatrogenic injury to the IANB.

## Data Availability

The datasets used and/or analyzed during the current study available from the corresponding author on reasonable request.

## Abbreviations

AL: Antilingula
AL_r_: Distance from the antilingula to the reference plane
AL_w_: Distance from the antilingula to the posterior border of the ramus
LG: Lingula
AMF: Anterior border of the mandibular foramen
PMF: Posterior border of the mandibular foramen
PMF_r_: Distance from the posterior border of the mandibular foramen to the posterior border of the ramus measured parallel to the reference plane
W: The narrowest width of the ramus in an antero-posterior direction measured parallel to the reference plane
CBCT: Cone beam computed tomography
CT: Computed tomography
IANB: Inferior alveolar neurovascular bundle
BSSRO: Bilateral sagittal split ramus osteotomy
IVRO: Intraoral vertical ramus osteotomy
ILO: Inverted-L osteotomy
